# Automated segmentation of multiple sclerosis lesions, paramagnetic rims, and central vein sign on MRI provides reliable diagnostic biomarkers

**DOI:** 10.1162/IMAG.a.932

**Published:** 2025-10-10

**Authors:** Fengling Hu, Zheng Ren, Luyun Chen, Alessandra M. Valcarcel, Jordan Dworkin, Brian Renner, Lynn Daboul, Carly M. O’Donnell, Elizabeth D. Verter, Abigail R. Manning, Kelly A. Clark, Eunchan Bae, Christina Chen, Carolyn Lou, Theodore D. Satterthwaite, Haochang Shou, Michel Bilello, Kunio Nakamura, Amit Bar-Or, Peter A. Calabresi, Leorah Freeman, Roland G. Henry, Erin E. Longbrake, Jiwon Oh, Matthew K. Schindler, Martina Absinta, Andrew J. Solomon, Nancy L. Sicotte, Daniel Ontaneda, Daniel S. Reich, Pascal Sati, Russell T. Shinohara

**Affiliations:** Penn Statistics in Imaging and Visualization Endeavor (PennSIVE), Department of Biostatistics, Epidemiology, and Informatics, Perelman School of Medicine, University of Pennsylvania, Philadelphia, PA, United States; Genentech Inc. A Member of the Roche Group, South San Francisco, CA, United States; Department of Neurology, Cedars-Sinai Medical Center, Los Angeles, CA, United States; Biomedical Imaging Research Institute, Cedars-Sinai Medical Center, Los Angeles, CA, United States; Translational Neuroradiology Section, National Institute of Neurological Disorders and Stroke, National Institute of Health, Bethesda, MD, United States; Department of Neurology, Brigham and Women’s Hospital, Boston, MA, United States; Pfizer Inc., Brooklyn, NY, United States; Department of Psychiatry, Perelman School of Medicine, University of Pennsylvania, Philadelphia, PA, United States; Penn-CHOP Lifespan Brain Institute, University of Pennsylvania, Philadelphia, PA, United States; The Penn Lifespan Informatics and Neuroimaging Center, Department of Psychiatry, Perelman School of Medicine, University of Pennsylvania, Philadelphia, PA, United States; Center for Biomedical Image Computing and Analytics (CBICA), Perelman School of Medicine, University of Pennsylvania, Philadelphia, PA, United States; Department of Radiology, Perelman School of Medicine, University of Pennsylvania, Philadelphia, PA, United States; Department of Biomedical Engineering, Lerner Research Institute, Cleveland Clinic, Cleveland, OH, United States; Department of Neurology, Perelman School of Medicine, University of Pennsylvania, Philadelphia, PA, United States; Department of Neurology, Johns Hopkins University School of Medicine, Baltimore, MD, United States; Department of Neurology, Dell Medical School, The University of Texas, Austin, TX, United States; Department of Neurology, University of California at San Francisco, San Francisco, CA, United States; Department of Neurology, Yale University, New Haven, CT, United States; Division of Neurology, St. Michael’s Hospital, University of Toronto, Toronto, ON, Canada; Department of Neurological Sciences, Larner College of Medicine, The University of Vermont, Burlington, VT, United States; Department of Biomedical Sciences, Humanitas University, Milan, Italy; IRCCS Humanitas Research Hospital, Milan, Italy; Mellen Center for Multiple Sclerosis, Cleveland Clinic, Cleveland, OH, United States

**Keywords:** multiple sclerosis, deep learning, lesion segmentation, paramagnetic rim lesions, central vein sign

## Abstract

Multiple sclerosis (MS) is characterized by central nervous system lesions detectable via MRI. Existing diagnostic criteria incorporate presence of white matter lesions, but specificity can be improved using MS-specific imaging biomarkers, including paramagnetic rim lesions (PRLs) and central vein sign (CVS). However, manual segmentation of lesions, PRLs, and CVS is time-consuming and subjective. We propose a fully-automated joint segmentation method called Automated Lesion, PRL, and CVS Analysis (ALPaCA). We trained ALPaCA using subject-level cross-validation on 47 adults with MS and 50 adults with radiological MS mimics. ALPaCA uses a voxel-wise lesion segmentation method to propose a large set of lesion candidates. Lesion candidates are input into a multi-contrast, multi-label 3D convolutional neural network as 3D patches to produce lesion, PRL, and CVS predictions. When multiple lesions exist within a patch, an attention mechanism identifies which lesion candidate to classify. At the lesion level, ALPaCA achieves cross-validation areas under the receiver operating characteristic curve (AUROCs) of 0.95, 0.91, and 0.87 for lesion, PRL, and CVS classification, outperforming previous methods (all p < 0.001). Correlations between subject-level ALPaCA lesion and PRL scores with manual counts are higher than those of previous methods (p < 0.001; p = 0.03). Subject-level ALPaCA PRL and CVS scores are highly associated with MS in logistic regressions, when controlling for age and sex (p < 0.001). ALPaCA allows for fully-automated simultaneous segmentation of MS lesions, PRLs, and CVS using clinically-feasible scans. These segmentations outperform existing methods at the lesion and subject level.

## 1 Introduction

Multiple sclerosis (MS) is a demyelinating, inflammatory disorder characterized by brain and spinal cord lesions detectable using MRI (Ma et al., 2022). MS lesions are used for diagnosis, prognosis, measuring disease burden, monitoring treatment response, and tracking disease progression (Rovira & León, 2008).

Current MS diagnostic criteria were developed to prioritize sensitivity in patients with common clinical presentations of MS, such as a relapsing-remitting course. When applied incorrectly, these criteria may result in high misdiagnosis rates (Solomon et al., 2016). This is especially true for neurological disorders presenting with MRI white matter lesions, referred to as radiological MS mimics. To better assess MS disease burden and minimize misdiagnosis, two diagnostic biomarkers that are pathologically specific for MS, namely paramagnetic rim lesions (PRLs) and the central vein sign (CVS), have been proposed as part of revisions to diagnostic criteria (La Rosa et al., 2022; Maggi et al., 2018; Maggi, Sati, et al., 2020). PRLs are chronic active lesions associated with more severe disability (Absinta et al., 2018, 2021; Hemond et al., 2022). On susceptibility-sensitive MRI, PRLs are characterized by a paramagnetic shift at the lesion periphery corresponding to iron-laden phagocytic cells (Dal-Bianco et al., 2017), while CVS is defined as the presence of a single vein running through the center of a white matter lesion (Sati et al., 2016). CVS lesions represent perivenular demyelination, a histopathological hallmark of MS (Ontaneda et al., 2021).

Despite the potential diagnostic benefits of lesion burden, PRLs and CVS, these biomarkers are not routinely assessed in clinical practice, since evaluating them is time-consuming, requires specialized training, and is subject to inter-rater variability (Absinta et al., 2018; La Rosa et al., 2022). Thus, a number of automated methods have been proposed (La Rosa et al., 2022). One method for lesion segmentation, Method for Inter-Modal Segmentation Analysis (MIMoSA), uses logistic regression on multi-contrast features to make voxel-wise lesion predictions (Valcarcel et al., 2018). Automated PRL (APRL) extracts radiomic features from susceptibility-based T2* echo planar imaging phase (EPIp) contrasts and makes predictions using these features with a random forest (Lou et al., 2021). For CVS detection, automated CVS (ACVS) employs 3D Frangi filters on EPI magnitude (EPIm) contrasts to identify vessel-like voxels within lesions and assess their centrality (Dworkin, Sati, et al., 2018).

Deep learning methods have been previously applied for lesion segmentation as well as PRL and CVS classification. For lesion segmentation, methods incorporating 3D U-Nets and convolutional neural networks have shown promise (La Rosa et al., 2020; Valverde et al., 2017). For PRLs and CVS, RimNet is a 3D convolutional neural network (CNN) that predicts whether patches from FLAIR and EPIp contrasts contain a PRL (Barquero et al., 2020), and CVSnet, another 3D CNN, predicts whether an EPIm patch contains a CVS (Maggi, Fartaria, et al., 2020). However, RimNet requires manual intervention to split confluent lesions, exclude very large lesions, and exclude patches containing multiple lesions—this limits its ease of use and ability to classify some important lesions. Meanwhile, CVSnet requires manual intervention to segment lesions, exclude ineligible lesions, and exclude patches containing multiple lesions (Maggi, Fartaria, et al., 2020). Additionally, CVSnet cannot produce voxel-level segmentations and is not publicly available. Notably, no method has been designed to capitalize on the mutual information between lesions, PRLs, and CVS in order to produce joint segmentations. This methodological gap may be partially attributable to the inherent challenge of collecting a single dataset where all participants have undergone suitable imaging for visualizing all three biomarkers, and manual labels for all biomarkers are available across these participants.

We propose an open-access, fully-automated deep learning method called Automated Lesion, PRL, and CVS Analysis network (ALPaCA). ALPaCA generates voxel-wise lesion candidates and jointly classifies them as typical lesions, PRLs, or CVS using a multi-contrast, multi-label, label-dependency, ensemble 3D CNN using T1-weighted, FLAIR, EPIm, and EPIp contrasts.

## 2 Materials and Methods

### 2.1 CAVS-MS dataset

We included 97 participants from the Central Vein Sign in Multiple Sclerosis Pilot Study (CAVS-MS Pilot) from the North American Imaging in MS Cooperative (NAIMS) (Daboul et al., 2024). MS diagnosis was adjudicated using 2017 McDonald criteria. As part of this criteria and reflecting the study period, individuals with clinically-isolated syndrome (CIS) or radiologically-isolated syndrome (RIS) were classified as not having MS; however, since recently proposed changes to these criteria may affect these classifications, sensitivity analyses for these classifications are performed. Institutional Review Boards approved the study. 3T 3D MRI sequences were collected, including T1-weighted MPRAGE (T1w), T2-weighted FLAIR, and T2*-weighted segmented EPI magnitude and phase (EPIm, EPIp). Inclusion criteria included: age 18 to 65, referral for suspicion of MS, and brain MRI demonstrating focal white matter T2 hyperintensities. Exclusion criteria included: use of DMTs, contraindications to MRI or gadolinium contrasts, and treatment with systemic corticosteroids within 4 weeks. Recruitment occurred between April 2018 and February 2020, and informed consent was obtained for all participants. Acquisition details were previously described (Daboul et al., 2024).

Gold-standard assessments included approximate central coordinates of lesions and rating of PRL and CVS status based on proposed NAIMS criteria, blinded to MS diagnosis (Sati et al., 2016). Lesions were labeled by one trained rater (LD, 4th-year medical student) who was trained to identify all lesions, regardless of lesion location or lesion size. PRLs were independently labeled by three trained raters (PS, medical physicist; BR, research associate; EDV; neurology resident), and disagreements were adjudicated by a fourth rater (MA; neurologist). CVS lesions were labeled by one trained rater (LD). All raters had access to all four sequences. Inter-rater reliability of CVS status has been previously evaluated at the subject level and showed moderate to substantial agreement (Daboul et al., 2024). Inter-rater reliability of PRL status was not assessed as PRLs were rated by consensus across raters.

Coordinates were originally identified for clinical research studies to uniquely refer to a given lesion; voxel-level segmentations were not generated for these studies. Thus, the locations of lesions, PRLs, and CVS were readily available from these studies, but exact lesion boundaries were not. Performance metrics for MIMoSA, APRL, ACVS, and ALPaCA were obtained by checking whether automated voxel-wise lesion predictions and their corresponding PRL and CVS statuses included the correct gold-standard coordinates.

Since each clinical research study had varying inclusion criteria, patterns of missing data in gold-standard coordinates differed across biomarkers—while a complete set of PRL coordinates was available for all 97 participants, a complete set of CVS coordinates was available for 92 participants, and coordinates of T2 lesions were complete for 58 participants. For participants with incomplete T2 lesion coordinates, some lesions were labeled during PRL or CVS identification, but unmarked lesion candidates could be either non-lesions or missing data. Additionally, CVS status could not be confidently assigned for 85 lesions labeled with “unsure” CVS status.

Missing data in the T2 lesions followed a missing completely at random structure at the subject level; as part of a clinical study on CVS, LD started labeling all CVS and T2 lesions for CAVS-MS participants, including both lesions eligible for CVS evaluation and lesions excluded from CVS evaluation by proposed NAIMS criteria. After completing full ratings for 58 participants, an interim meeting between LD and her advisors led to a decision that labeling of lesions excluded from CVS evaluation was not relevant for the clinical CVS study. Thus, only lesions meeting NAIMS CVS inclusion criteria were labeled for the remaining 39 participants, such that missing T2 lesions followed a missing at random structure with missingness conditional on CVS inclusion criteria as well as positive PRL status. Missing data in the CVS followed a missing at random structure conditional on slightly decreased EPIm image quality, as stringent image quality metrics were applied in the clinical CVS study such that five subjects were excluded from analysis (Daboul et al., 2024). However, quality of these images was deemed to be acceptable for this study for the purposes of increased sample size and generalizability. Additionally, since CVS labels were not available for these subjects, we thought the risk of introducing bias due to these images was minimal.

For MS participants, the final dataset contained manual coordinates of 2380 lesions, 122 PRLs, and 534 CVS. For non-MS participants, coordinates for 3331 lesions, 26 PRLs, and 130 CVS were identified. Subject-level counts for lesions, PRLs, and CVS were available for 94, 97, and 92 participants. This included 36 participants where total counts of subject-level T2 lesions were available, but coordinates were not fully available for those lesions. For ALPaCA training, the above sources of incomplete or unsure labels were included and treated as missing data. However, results are reported only on the set of complete data.

### 2.2 Image preprocessing

The following image preprocessing was performed: 1) N4 bias correction (all sequences), 2) rigid registration to EPIm space using Advanced Normalization Tools (ANTs) Symmetric Normalization (SyN) with Lanczos windowed sinc interpolation (T1w, FLAIR), 3) skull stripping using Multi Atlas Skull Stripping on T1 (applied to all sequences), and 4) intensity normalization to a standard normal distribution (all sequences) (Avants et al., 2008; Doshi et al., 2013; Tustison et al., 2010). Phase unwrapping was performed using a Laplacian-based unwrapping pipeline (Absinta et al., 2016; Dewey, 2022). EPIp was not registered to EPIm space since EPIp and EPIm were collected from the same acquisition and were, therefore, already in the same space.

### 2.3 ALPaCA architecture

The ALPaCA method comprises three independent components which together allow for voxel-wise segmentations of lesions, PRLs, and CVS. Briefly, the first step identifies a set of proposal voxels that appear to exist within T2 lesions. Then, the second step seeks to cluster these voxels into a partition of distinct lesion candidates. Finally, these lesion candidates are classified as lesions, PRLs, or CVS, or discarded as false positives.

To produce proposal voxels, ALPaCA uses an automated voxel-wise segmentation algorithm, MIMoSA, since voxel-wise segmentations were unavailable to train a new segmentation neural network (Valcarcel et al., 2018). For this step, MIMoSA segmentations are liberally thresholded at 0.05 so masked voxels are sensitive, but not specific—this approach produces a large set of potential lesion candidates from which ALPaCA can subsequently filter out false positive candidates. Since a connected MIMoSA segmentation can comprise multiple confluent lesions, confluent clusters are partitioned into distinct lesion candidates using a previously-proposed automated Hessian-based approach (Dworkin, Linn, et al., 2018). This method identifies distinct lesion centers via peaks of maximum lesion probabilities and labels non-center voxels by their closest lesion center.

**Fig. 1.**
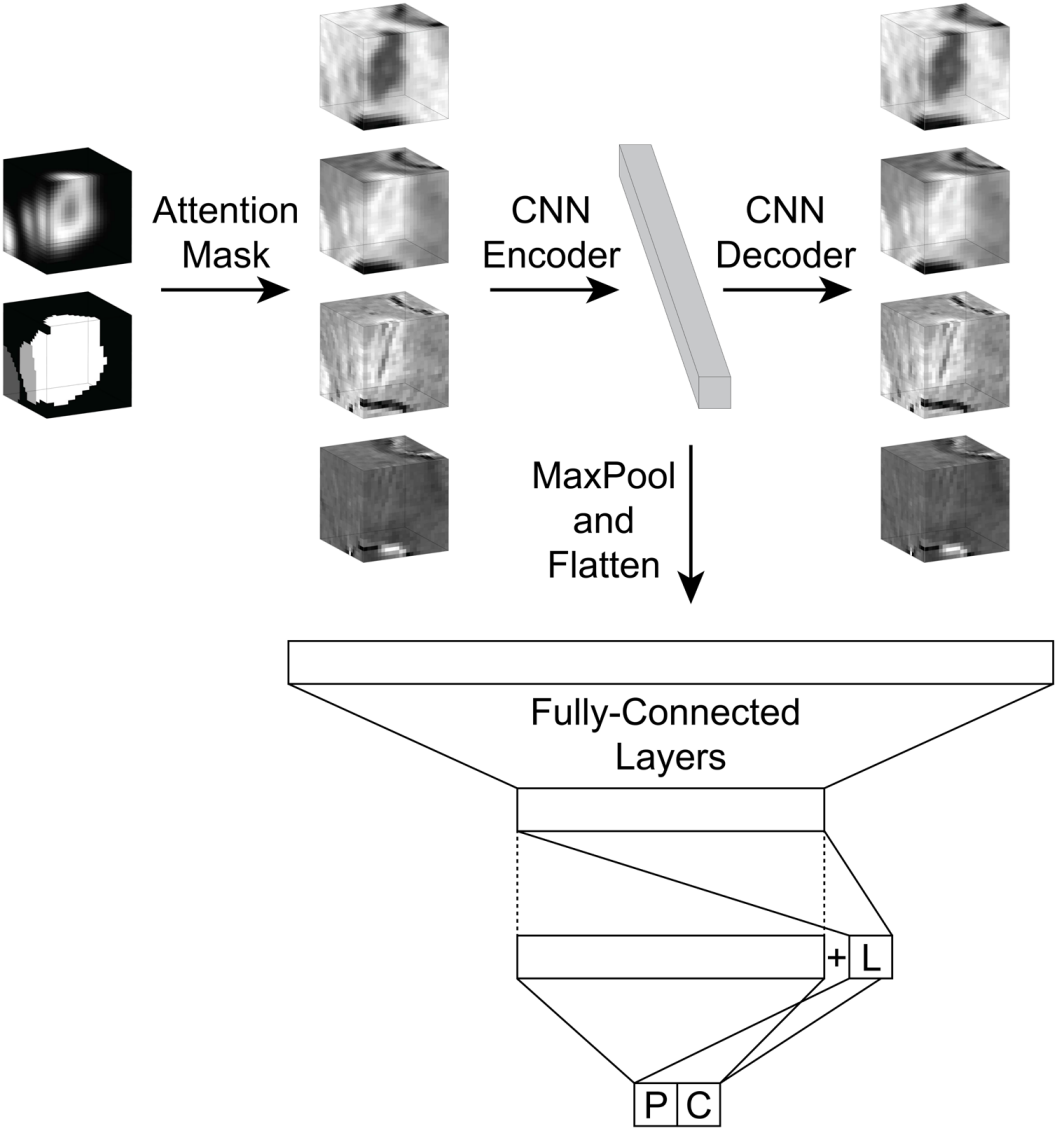
ALPaCA incorporates an automated MS-specific lesion segmentation algorithm to propose lesion candidates and allow for attention masking (Left). Multi-contrast patches are built around these candidates and passed through a 3D CNN. Encoder parameters are pretrained using an autoencoder architecture (Center and Right). The decoder is then discarded and the autoencoder latent space (Center) is attached to the fully-connected, multi-label, dependency predictor network in order to jointly predict lesion, PRL, and CVS status (Bottom). Outputs are produced from the predictor network in the last two layers. First, lesion status (L) is predicted in the second-to-last layer and then concatenated with a copy of the above layer, where dashed lines represent duplication. Then, this second-to-last layer is used to predict PRL (P) and CVS (C) status in the final layer.

Lesion candidates are passed to ALPaCA to classify using a multi-contrast, multi-label, label-dependency 3D patch CNN (Fig. 1). The network uses 24 x 24 x 24 voxel patches around lesion candidates from the four above contrasts, resulting in a 4 x 24 x 24 x 24 tensor input. To overcome the need for manual intervention, patches are not required to be centered around candidates, cover the entire candidate, or include only one candidate. The encoder consists of five 3 x 3 x 3 convolutional layers with sizes: 4, 64, 128, 512, and 2048. All layers are followed by batch normalization and ReLU activation; the last three layers are additionally down-sampled using 2 x 2 x 2 max pooling. For pre-training, the decoder consists of the corresponding inverse layers and sizes. For prediction, the predictor is fully connected with ReLU activation, 20% dropout, and the following layer sizes: 2048, 512, 128, 129, and 2. The layer with 129 nodes is created by duplicating the layer with 128 nodes and concatenating an additional node containing the lesion prediction with sigmoid activation. Sigmoid activation is also used in the final layer. Ultimately, the network outputs a three-label prediction for lesion, PRL, and CVS status, where each prediction ranges from 0 to 1.

To improve generalizability and prevent overfitting, we performed unsupervised pretraining using spatial and intensity data augmentation. Patches were sampled from these augmented brains, and attention masks were created for each patch. Attention masks specified which candidate should be classified if multiple candidates were present in a patch and which regions of the patch were inside the given lesion candidate. This step allowed ALPaCA to overcome the limitations of RimNet and CVSnet that naturally arise due to a patch-based approach.

Then, the convolutional decoder was discarded, and the encoder was attached to the fully-connected predictor. Binary cross-entropy loss across the three outputs was used, where the target output was based on overlap of the lesion candidate with manual central coordinates. Missing data in the coordinates were accounted for by weighting the relevant loss component to 0, such that the network was not penalized for any prediction since the ground truth was unknown.

Notably, since the set of PRLs and CVS is contained within the set of lesions (i.e., a PRL or CVS must also be a lesion), ALPaCA incorporates label dependencies by first predicting lesion status, then using this prediction for PRL and CVS status predictions (Fig. 1). Thus, predictions incorporate domain-specific relationships to borrow across tasks and augment available information.

To make predictions using ALPaCA, 25 randomly-sampled patches from each lesion candidate are independently run through the network, and their predictions are averaged to obtain the final lesion-level probabilistic predictions for each output. Intuitively, this ensemble procedure allows for better coverage of pertinent lesion features and increased robustness such that lesions do not have to be centered or fit entirely in a patch.

#### 2.3.1 ALPaCA training

We used 10-fold subject-level cross-validation such that, for each of 10 models trained, approximately 90% of the subjects were included in the training set while the remaining subjects were used for cross-validation. In a given fold, patches were sampled from each subject after subjects were assigned to either the training set or validation set, such that all patches from a given subject ended up in the same set with no crossover to the other set. Results shown below are based on cross-validation performance metrics such that each subject is evaluated using the model that did not train on that subject. No external test set was used.

Preprocessed brains are spatially augmented via random flips, random rotation, and random rescaling from 0.66x to 1.5x with linear interpolation, and intensity augmented via random contrast changes using Gamma correction with log-gamma values from -0.5 to 0.5 (Pérez-García et al., 2021). Patches are sampled from these augmented brains, with oversampling of patches containing lesion candidates. In convolutional encoder pretraining, the central voxel of a patch is sampled based on its MIMoSA probability prediction. In joint training, the central voxel is sampled such that, compared to false positive lesion candidates, true lesions, PRLs, and CVS lesions are each sampled three times more often. Within each epoch, 50 patches are randomly sampled from each participant in the training set, shuffled along with patches from other participants, and passed through the CNN in mini-batches of 64 patches. We pretrained the encoder for 50 epochs with L2 loss using a convolutional autoencoder. Once connected to the predictor, the joint network was trained for 100 epochs with the same augmentation and attention masking as in pretraining.

For attention masking, a single lesion candidate of interest is randomly chosen from all candidates in the patch. For the EPIm contrast, which is useful for identifying central veins, voxels outside of the candidate are zeroed out, and non-surface voxels of the candidate are emphasized by multiplying their values by 2—since convolutional filters are translation invariant, this masking procedure allows ALPaCA to ignore veins outside of the candidate as well as discount non-central veins within the candidate. When multiple lesion candidates are present in the patch, non-EPIm voxels located within other lesion candidates are dimmed by multiplying their values by 0.1. This allows ALPaCA to identify which lesion candidate to classify but still maintains context from other candidates.

For loss function weighting, a weighted mean squared error (MSE) loss function was used in pretraining, where reconstruction loss for lesion candidate, EPIm, and EPIp voxels were upweighted 4x. Upweighting prioritizes extraction of lesion-specific as well as EPIm- and EPIp-specific features, since these contrasts have lower signal-to-noise ratio compared to T1 and FLAIR. To deal with missing data in joint training, for participants where lesion or CVS coordinates were not available, the relevant loss component was given a weight of 0, allowing the model to still learn from non-missing data for those participants. When CVS status was marked as “unsure,” the candidate’s CVS loss was weighted to 0.

Patch size, training epochs, mini-batch size, and learning rate were chosen a priori. Patch size of 24 x 24 x 24 voxels was chosen based on visual inspection of whether lesions could be well-classified by eye in various patch sizes and based on similar patch sizes in RimNet and CVSnet. A total of 150 training epochs was chosen such that training was completed after loss functions plateaued. Mini-batch size was chosen based on memory limitations and based on expert recommendations. The Adam optimizer was used for all epochs with a default learning rate of 0.001 (Kingma & Ba, 2017). No additional hyperparameter optimization was performed, as standardization of the input patches to the appropriate scale was thought to be sufficient. PyTorch version 1.10.2 was used. Overall, training, which included data augmentation and pretraining, took approximately 24 h. On one core of an Intel Xeon CPU (E5-2630 v3 @2.40GHz), inference on one patch takes less than 1 s.

### 2.4 Comparison methods

ALPaCA predictions for lesion, PRL, and CVS status are evaluated against segmentations produced by MIMoSA, APRL, and ACVS, respectively. RimNet and CVSnet are not included as comparisons since RimNet and CVSnet are not fully automated, and CVSnet is not publicly available. MIMoSA, APRL, and ACVS and their implementation in this manuscript are briefly described below. For these models, image pre-processing is performed as recommended in the original manuscripts.

MIMoSA is a voxel-wise method that first extracts a number of local features from the neighborhood of each voxel for a given subject. We used the pre-trained model using T1 and FLAIR images, which extracts local information from T1 and FLAIR images as well as from a contrast describing the intermodal relationship between these images. Then, MIMoSA runs logistic regression across the local features for each voxel, producing voxel-wise lesion probabilities. In this manuscript, for each lesion candidate, its overall MIMoSA prediction is defined as the maximum MIMoSA prediction of all voxels in that lesion candidate. This serves to simulate the thresholding that would be performed if MIMoSA were to be used on the images—for any given threshold, any lesion candidate with at least one voxel with MIMoSA prediction higher than the threshold would be preserved in the final segmentation.

APRL makes predictions on MIMoSA-segmented lesion candidates using a radiomics-based approach (Lou et al., 2021). First, APRL thresholds MIMoSA at a value of 0.2 in order to identify lesions for PRL classification. This threshold serves to increase specificity of T2 lesions since APRL cannot rule out false positive T2 lesions. As a result, lesion candidates with MIMoSA probabilities under 0.2 are, by default, assigned PRL predictions of 0 by APRL. Once MIMoSA segmentations are produced, APRL extracts 44 first-order, manually-selected radiomics features which serve to describe the average and spread of the intensities, the shape of the distribution of intensities, and the diversity of intensities within a given MIMoSA lesion segmentation. Finally, a random forest model is trained on these radiomics features in order to predict PRL status of the MIMoSA-based lesion segmentation.

ACVS makes predictions using statistical-based permutation analyses of 3D Frangi filter outputs (Dworkin, Sati, et al., 2018). First, using the overall EPIm images, 3D Frangi vesselness filters are applied to identify vessel-like structures in the brain parenchyma. To obtain lesion segmentations for classification and similarly to APRL, MIMoSA voxel-wise predictions are produced and thresholded at 0.2 to identify high-specificity lesion candidates for CVS classification. As with APRL, lesion candidates with MIMoSA probabilities under 0.2 are assigned CVS predictions of 0. Once lesion segmentations are obtained, periventricular lesions are filtered out according to proposed NAIMS criteria, and confluent lesions are split using the Hessian-based approach described above. Then, lesion segmentations are cross-referenced with corresponding Frangi filter vessel in order to calculate a “vessel centrality score” such that lesion segmentations have a higher score when identified vessels are far from the lesion boundary. Since the distribution of possible vessel centrality scores may differ based on specific lesion or vessel shape, the empiric distribution of lesion-specific vessel centrality scores is produced via permutation, and an overall lesion-specific CVS probability is defined as the probability of seeing a vessel centrality score as high, or higher, than that observed in the lesion.

### 2.5 Statistical analysis

ALPaCA performance compared to both manual labels and automated segmentations by MIMoSA, APRL, and ACVS were evaluated at the level of both lesions and subjects. Lesion-level performance is evaluated via receiver operating characteristic curves (ROC) and corresponding pairwise DeLong’s tests to test for difference in area under the ROC (AUROC). Missing data were excluded from the ROCs to prevent bias.

Lesion-level segmentations are also qualitatively evaluated via thresholding of the ROC curve based on Youden’s J of the training ROC curve across cross-validation folds. Youden’s J threshold was used in order to maximize the objectivity of these examples. This qualitative analysis was performed on 20 randomly selected subjects. For each subject, up to three ALPaCA false positives and three false negatives were randomly selected for each of the lesion, PRL, and CVS prediction tasks, such that a total of up to 18 examples could be sampled from the subject. Fewer than 18 examples could be sampled if fewer than three false-positive or false-negative predictions existed for any of the three tasks. For each example, an axial, coronal, and sagittal slice from the center of the lesion was shown to one rater (FH, medical student, and PhD imaging scientist) who assessed whether, based on the observed slices, the example was more likely due to an ALPaCA error or a mislabeled gold standard. NAIMS criteria were used for CVS lesions; to align with the ALPaCA framework, exceptions were made for confluent lesions that appeared separable from nearby lesions. Regardless of the assessment, the rater attempted to categorize the errors based on lesion features.

Additionally, to provide some degree of interpretability for CVS predictions, associations between lesion-level ALPaCA CVS predictions and manually-identified lesion features were evaluated via multivariate linear models as well as qualitatively.

For subject-level comparisons, lesion predictions were aggregated within subjects for manual labels, ALPaCA predictions, and predictions from MIMoSA, APRL, and ACVS. Threshold-invariant subject-level ALPaCA lesion scores were obtained by adding lesion predictions across all candidates within subjects. Threshold-invariant subject-level PRL percentage scores were estimated by averaging PRL predictions across candidates, weighted by their lesion predictions and multiplying by 100. Subject-level CVS percentage scores were calculated identically to PRL percentage scores. The same procedure was used to generate subject-level manual label biomarkers and biomarkers from MIMoSA, APRL, and ACVS. Threshold-invariant lesion, PRL, and CVS scores were used instead of post-threshold biomarker counts since counts are subject to high variance based on chosen threshold, as well as potential bias based on how optimal thresholds are chosen. Then, associations between these aggregated predictions and MS diagnosis were assessed using univariate logistic regression models. Additionally, predictive value of these aggregated predictions was evaluated by fitting multivariate logistic generalized additive models (GAMs) using default-setting smooth terms (Hastie & Tibshirani, 1986). These models are described using Akaike Information Criterion (AIC), ROC curves, and DeLong’s test.

R version 4.3.0 was used for all statistical analyses, and the pROC and mgcv packages were used for producing ROCs and GAMs, respectively.

## 3 Results

### 3.1 Lesion-level performance

CAVS-MS Pilot participant demographics are shown in Table 1. Overall, in MS participants, 5703 lesion candidates were proposed via liberal MIMoSA thresholding with lesion splitting. These lesion candidates captured 1700 manually identified lesions, 111 PRLs, and 424 CVS (71.4%, 91.0%, and 79.4% of total manual labels in MS participants, respectively). In non-MS participants, 4808 lesion candidates were proposed, capturing 919 manually identified lesions, 20 PRLs, and 100 CVS (27.6%, 76.9%, and 76.9% of total manual labels in non-MS participants, respectively). Notably, MS lesions, PRLs, and CVS were much more likely to be included for ALPaCA consideration compared to non-MS T2 lesions. Additionally, chi-squared tests showed T2 lesions and PRLs were detected at significantly higher rates in MS participants compared to non-MS participants (p <0.001 and p = 0.04). No significant difference in CVS detection between the two groups was observed (p = 0.53). Missed non-MS typical lesions tended to be small and punctate on visual inspection.

**Table 1.** Participant demographics, split by diagnosis.

Demographics	MS participants (n = 47)	Non-MS participants (n = 50)
Age	39 [33.5, 52]	48 [39.5, 55.8]
Female	30 (64%)	41 (82%)
Expanded disability status scale	1.5 [0.25, 2]	1.25 [0.25, 2]
Lesion characteristics
Lesion count	36 [20, 71]	40 [20, 106]
PRL count	1 [0.5, 4]	0 [0, 0]
CVS count	9 [3, 15]	2 [0, 3]
Median [IQR]; n (%)		

Individuals with radiologically isolated syndrome (RIS) or clinically isolated syndrome (CIS) were included in the non-MS group.

**Fig. 2.**
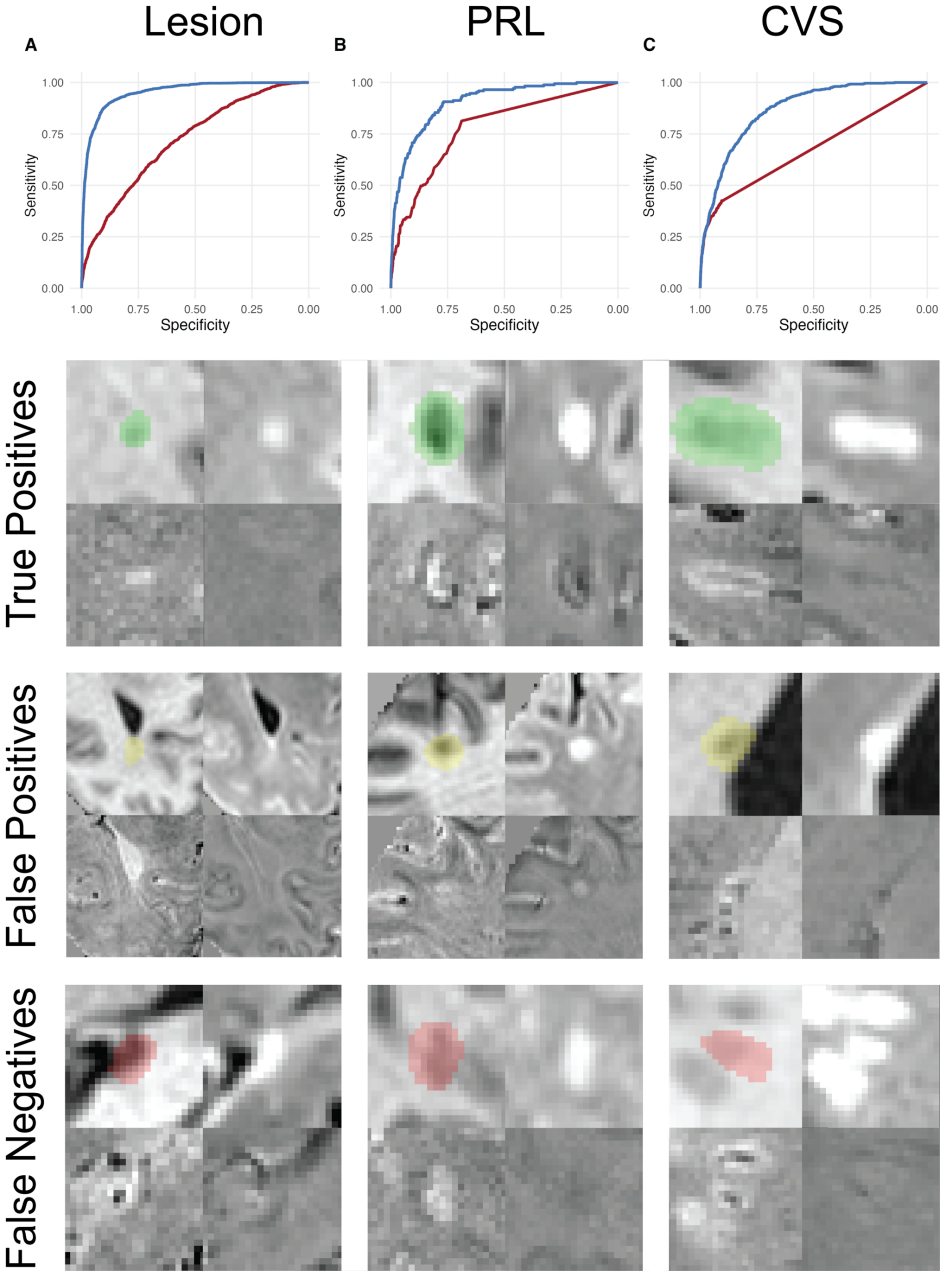
(A) ALPaCA lesion ROC plot compared to MIMoSA. (B) ALPaCA PRL ROC plot compared to APRL, (C) ALPaCA CVS ROC plot compared to ACVS. ALPaCA ROCs are shown in blue, and MIMoSA, APRL, and ACVS ROCs are shown in red. Specificity is shown on the X axis with higher specificity on the left. Sensitivity is shown on the Y axis. Missing data were excluded from ROC plots. Bottom: Representative ALPaCA true-positive, false-positive, and false-negative predictions for lesions, PRLs, and CVS. Clockwise from top left, each example shows the lesion candidate on T1w, FLAIR, EPIp, and EPIm. Voxel-wise segmentations are shown on the T1w contrast. The PRL false positive example is shown at 0.5x magnification compared to the others to better illustrate its proximity to the gray matter boundary.

ALPaCA achieved areas under the ROC (AUROC) of 0.95 (95% CI: [0.947, 0.957]), 0.91 (95% CI: [0.884, 0.927]) and 0.87 (95% CI: [0.854, 0.881]) for lesions, PRLs, and CVS, respectively (Fig. 2). Meanwhile, the AUROCs from maximum MIMoSA probabilities, APRL, and ACVS were 0.72 (95% CI: [0.704, 0.729]), 0.78 (95% CI: [0.745, 0.814]), and 0.67 (95% CI: [0.651, 0.696]). ALPaCA AUROCs were higher than the corresponding AUROCs from MIMoSA, APRL, and ACVS by DeLong’s test (all p < 0.001). Sensitivities and specificities at Youden’s J are provided in Table 2.

**Table 2.** Sensitivities and specificities of ALPaCA, MIMoSA, APRL, and ACVS when thresholded at Youden’s J.

Method	Threshold	Sensitivity	Specificity
ALPaCA Lesion	0.789	88.6%	89.2%
ALPaCA PRL	0.106	78.9%	84.7%
ALPaCA CVS	0.212	83.6%	74.1%
MIMoSA	0.172	70.3%	60.7%
APRL	0.001	81.3%	68.9%
ACVS	0.044	42.6%	90.5%

Additional thresholding values prioritizing either sensitivity or specificity can be used and are provided in the R package.

### 3.2 Qualitative lesion-wise analysis

Representative examples of ALPaCA voxel-wise segmentations are shown in Figure 2. In the qualitative analysis, lesion, PRL, and CVS predictions identified to be false positives or false negatives were assessed to evaluate whether these predictions represented true ALPaCA errors or mislabeled lesion candidates in the gold standard (Table 3). Additionally, the identified misclassifications were reviewed for common explanatory themes in order to identify which settings led to the most errors.

**Table 3.** Qualitative analysis of ALPaCA misclassifications for lesion, PRL, and CVS predictions.

		Common misclassification themes				
Lesion	Totals	Peri-ventricular	Juxta-cortical	Confluent		Other
ALPaCA +/manual –	58					
Reassessed +	46	15	1	9		21
Reassessed –	12	10	0	0		2
ALPaCA –/manual +	34					
Reassessed +	28	8	2	3		15
Reassessed –	6	6	0	0		0
PRL	Totals	Peri-ventricular	Juxta-cortical	Confluent	Pseudo-rim	Other
ALPaCA +/manual –	49					
Reassessed +	0	0	0	0	0	0
Reassessed –	49	23	14	0	2	9
ALPaCA –/manual +	5					
Reassessed +	5	1	0	2	0	2
Reassessed –	0	0	0	0	0	0
CVS	Totals	Peri-ventricular	Multiple vessels	Confluent	Faint central linearity	Other
ALPaCA +/manual –	60					
Reassessed +	11	2	0	0	4	5
Reassessed –	49	5	16	1	8	19
ALPaCA –/manual +	15					
Reassessed +	11	1	0	1	1	8
Reassessed –	4	1	1	0	1	1

Common reasons for misclassification were assessed.

Visually, lesions identified to be false positives tended to be true lesions missed by manual raters, confluent lesions identified by manual raters where only one lesion was labeled, or thin line periventricular T2 hyperintensities, including periventricular caps. False-negative lesions tended to occur near ventricles. False-positive PRLs tended to occur near the gray matter boundary or near ventricles as locally, the gray matter boundary or ventricle border is a hypointense surface on EPIp similar to a paramagnetic rim. False-negative PRLs tended to correspond to lesions with faint rims or were due to incorrect splitting of non-confluent lesions. False-positive CVS predictions tended to be lesions with veins that met exclusion criteria, such as periventricular lesions containing deep medullary veins, large lesions with multiple veins, or lesions with veins in a confluent cluster. False-negative CVS predictions tended to occur for smaller CVS lesions. Some false-positive and false-negative CVS lesions were deemed to be true positives and true negatives, respectively, on qualitative reassessment. Notably, these discrepancies, especially in the case of CVS assessment, could be due to viewing of lesions on only one slice each from axial, coronal, and sagittal planes which could preclude observation of additional vessels or discrimination of faint CVS and noise.

### 3.3 CVS interpretability

**Fig. 3.**
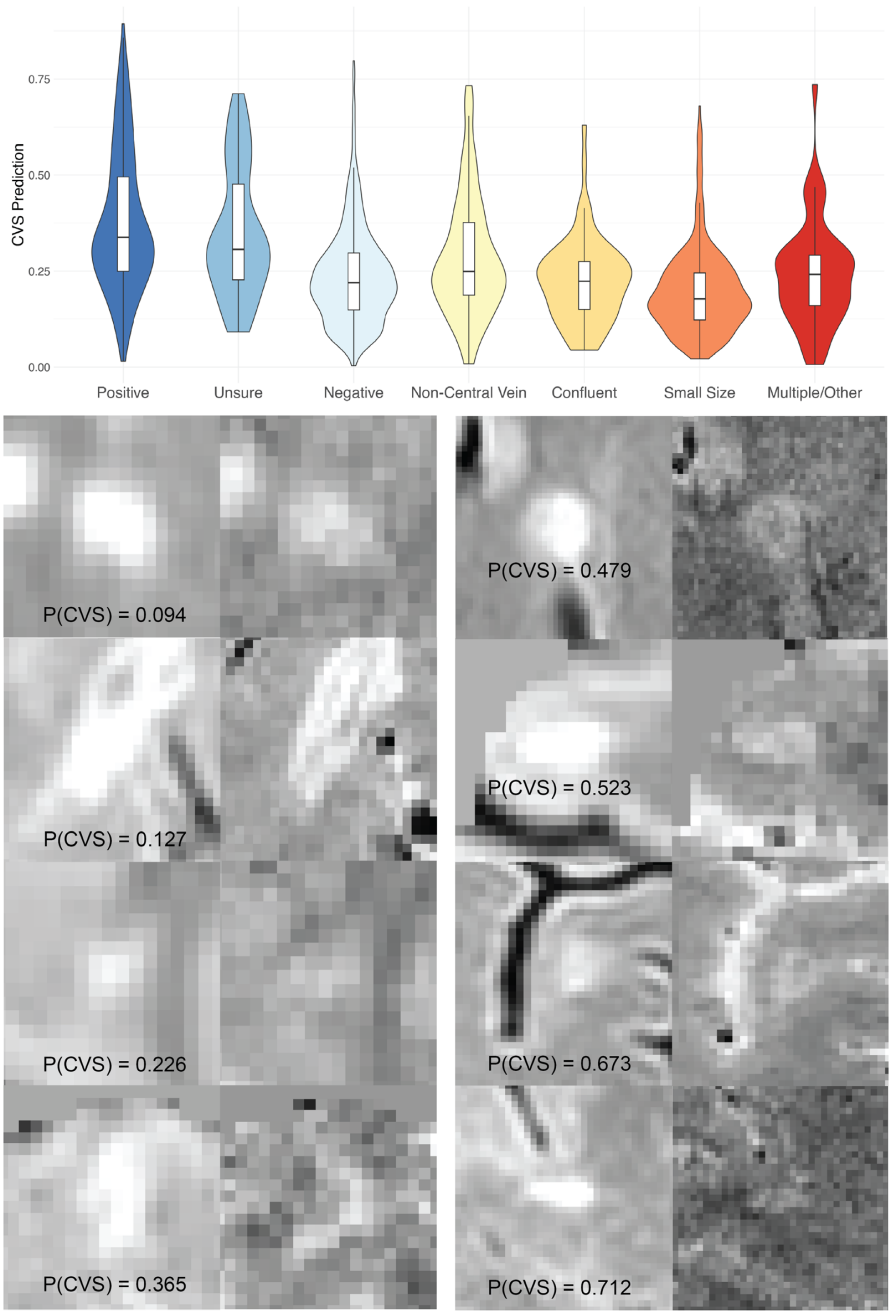
Top: Violin plots and boxplots of ALPaCA CVS predictions for manually-labeled CVS-positive, CVS-unsure, and CVS-negative lesions as well as four subcategories of lesions meeting exactly one CVS exclusion criteria or multiple CVS exclusion criteria. Bottom: Randomly-sampled examples of CVS-unsure lesions and corresponding ALPaCA predictions on FLAIR (left) and EPIm (right) sequences. Images are taken from the axial slice that most illustrates a potential central vein.

We explored how ALPaCA CVS predictions correlated with manually identified lesion features (Fig. 3, Top). Many non-CVS lesions were noted to have met proposed NAIMS exclusion criteria, including: diameter under 3 mm in the shortest axis; status as a confluent lesion; or presence of non-central veins such as eccentric, multiple, or branching veins (Sati et al., 2016). We used these annotations to fit a multivariate logistic regression model where ALPaCA CVS prediction was the outcome, and CVS-positive status, CVS-unsure status, small diameter, presence of non-central veins, and confluence were covariates. No similar annotations were available for typical lesions or PRLs.

540 out of the 1850 CVS-negative lesions were noted to have met exclusion criteria. On average, CVS-positive and CVS-unsure lesions were assigned higher predictions than CVS-negative lesions (β = 0.14, p < 0.001 and β = 0.11, p < 0.001). Lesions containing non-CVS veins also received higher predictions (β = 0.04, p < 0.001), while small lesions received lower predictions (β = -0.04, p < 0.001). These findings suggest ALPaCA correctly incorporates vein and size features into CVS predictions. Finally, confluent lesions did not have significantly different CVS predictions compared to CVS-negative lesions, which is consistent with the fact that ALPaCA splits confluent lesions before making predictions.

Investigating the relationship between CVS-unsure lesions and ALPaCA predictions, we found ALPaCA produces bimodal predictions for CVS-unsure lesions, predicting CVS probabilities in the high mode for 23 unsure lesions and CVS probabilities in the low mode for 62 unsure lesions. This suggests that ALPaCA identifies lesions assigned with CVS-unsure status to be a mixture of CVS and non-CVS lesions. Four randomly selected CVS-unsure lesions from each of the low and high mode are shown via FLAIR and EPIm images, along with their ALPaCA CVS prediction (Fig. 3, Bottom).

### 3.4 Subject-level performance

We aggregated lesion predictions within participants to assess how biomarkers based on manual labels, ALPaCA, and each of MIMoSA, APRL, and ACVS performed at predicting MS diagnosis. Analyses were performed in the 92 participants with complete manual counts. These resulted in the following threshold-invariant subject-level ALPaCA lesion scores (Median [IQR]) (MS: 60.0 [30.4, 104.4]; non-MS: 37.8 [18.0, 72.7]), PRL percentage scores (MS: 11.0 [6.1, 13.8]; non-MS: 3.3 [1.6, 6.3]), and CVS percentage scores (MS: 23.0 [20.2, 28.0]; non-MS: 16.8 [12.7, 22.7]). Similar scores were obtained from manual labels as well as MIMoSA, APRL, and ACVS.

**Fig. 4.**
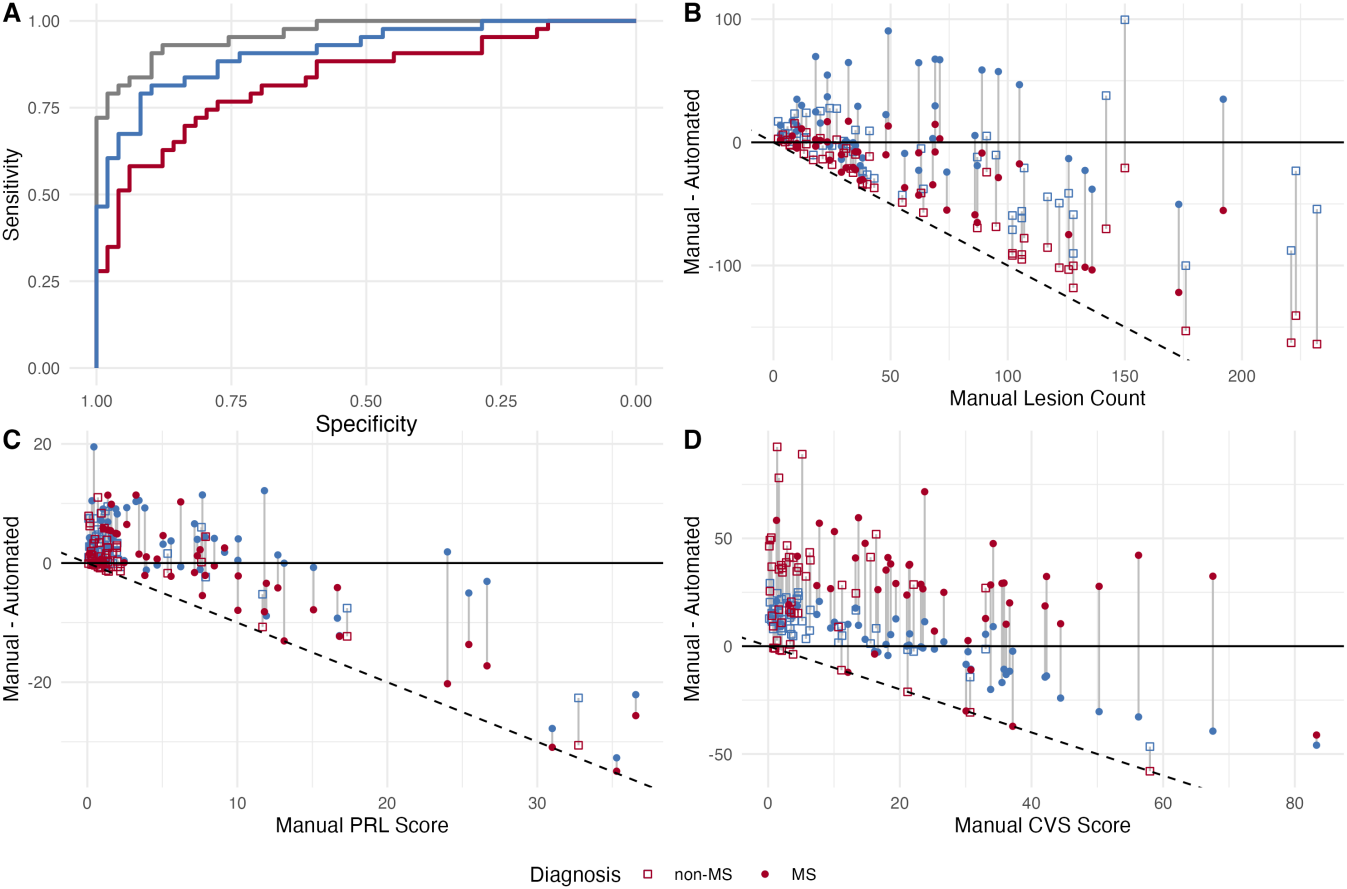
(A) ROCs for prediction of MS diagnosis with lesion, PRL, and CVS scores using logistic regression GAMs. The manual label ROC is shown in light blue, ALPaCA ROC is shown in dark blue, and the ROC using MIMoSA, APRL, and ACVS scores is shown in red. Specificity is shown on the X axis with higher specificity on the left. Sensitivity is shown on the Y axis. (B) Bland-Altman plot of ALPaCA lesion counts vs. manual lesion counts. (C) Bland-Altman plot of ALPaCA PRL scores vs. manual PRL scores. (D) Bland-Altman plot of ALPaCA PRL scores vs. manual PRL scores. ALPaCA scores are shown in dark blue, and MIMoSA, APRL, and ACVS scores are shown in red. Gray vertical lines represent that the points are different methods’ predictions for the same subject. The solid black line represents exact agreement between an automated score and manual score. The dotted black line represents the lowest bound for a point on the graph, which occurs if an automated method predicts a score of 0.

Pearson correlations between ALPaCA biomarker scores and corresponding manual biomarker scores were as follows: lesion score = 0.74 (95% CI: [0.64, 0.82]), PRL score = 0.45 (95% CI: [0.27, 0.60]), and CVS score = 0.45 (95% CI: [0.27, 0.60]). Correlations between each biomarker score from MIMoSA, APRL, and CVS and the corresponding manual biomarker counts were as follows: lesion score = 0.61 (95% CI: [0.47, 0.73]), PRL score = 0.21 (95% CI: [0.00, 0.40]), and CVS score = 0.45 (95% CI: [0.14, 0.50]). ALPaCA lesion scores and PRL scores were significantly more highly correlated with manual biomarkers than MIMoSA lesion counts and APRL scores by Meng’s z test for difference between two dependent correlations (p < 0.001; p = 0.03). ALPaCA CVS scores were not more highly correlated with manual CVS scores than ACVS scores (p = 0.26). Additionally, Bland-Altman plots showed that, when compared to manual biomarkers, ALPaCA scores were qualitatively more closely aligned and had less spread than scores from MIMoSA, APRL, and ACVS (Fig. 4). Additionally, the Bland-Altman plots illustrated that MIMoSA, APRL, and ACVS occasionally predicted lesion, PRL, and CVS scores near zero, even for subjects with high manual scores.

**Fig. 5.**
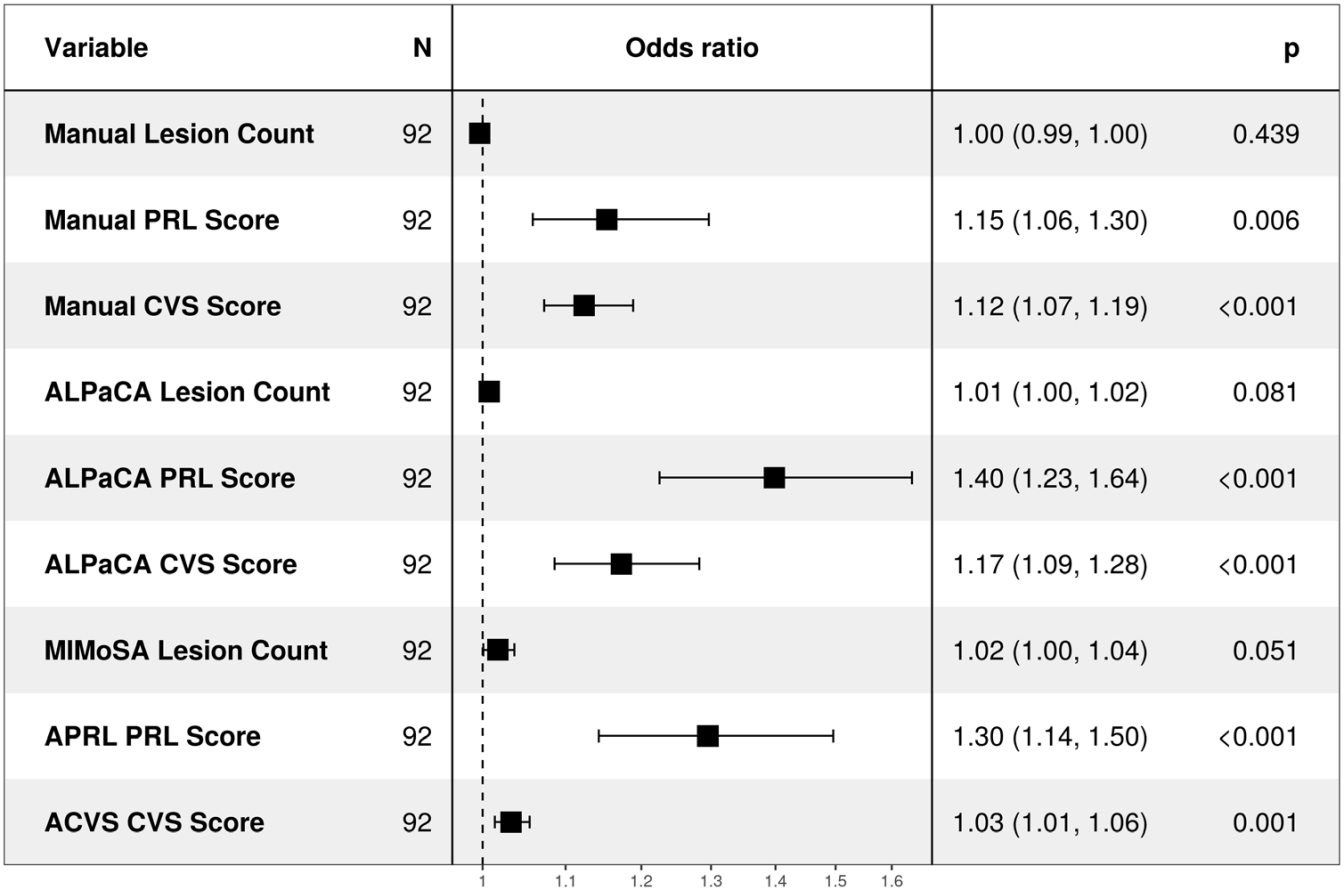
Forest plot of univariate odds ratios with respect to MS diagnosis in models where age and sex were controlled for. 95% confidence intervals are provided. Odds ratios are shown on a logarithmic axis.

To assess diagnostic associations, we fit univariate logistic regression models using each biomarker while controlling for age and sex. Univariate models were used since biomarkers within a subject tended to be highly collinear and effect sizes could not be well estimated in multivariate models. These analyses revealed that PRL and CVS scores obtained from manual labels, ALPaCA predictions, and above previous method predictions were all highly associated with MS diagnosis (Fig. 5). Additionally, ALPaCA and APRL PRL scores had comparable effect sizes to manual PRL scores, and ALPaCA CVS scores had a comparable effect size to manual CVS scores. Meanwhile, subject-level lesion count was not associated with MS diagnosis across for any analysis, reflecting the specificity of PRL and CVS biomarkers over lesion count.

Finally, to assess diagnostic performance, we fit multivariate logistic regression GAMs for lesion, PRL, and CVS scores for each set of subject-level biomarkers. These models also include linear terms for age and sex. The manual label model, ALPaCA model, and previous method model used 14.62, 11.57, and 7.75 total degrees of freedom. They had corresponding AICs of 74.3, 91.8, and 107.3, with lower AIC representing better fit. Predictions of MS diagnosis using these three models resulted in AUROCs of 0.963 (95% CI: [0.931, 0.995]), 0.912 (95% CI: [0.854, 0.970]), and 0.831 (95% CI: [0.746, 0.916]). DeLong’s test showed the manual label model had significantly higher AUROC than the previous method model (p = 0.001). Meanwhile, pairwise comparisons showed the ALPaCA model did not have significantly different AUROCs than either the manual label or previous method model (p = 0.10, p = 0.07). Overall, these results suggest that subject-level ALPaCA biomarkers may be more comparable to manual labels biomarkers in the context of disease prediction when compared to biomarkers derived from MIMoSA, APRL, and ACVS. Sensitivity analyses reclassifying individuals with RIS or CIS into the MS group as well as analyses excluding these individuals are included in the Supplemental Materials.

## 4 Discussion

We propose ALPaCA, a fully automated algorithm for segmenting lesions and MS-specific biomarkers with high sensitivity and specificity. ALPaCA is trained on a set of clinical research datasets with heterogeneous, but identifiable, patterns of missingness, including lack of voxel-wise segmentations and incomplete data within some subjects. In this setting, we demonstrate that effective training can be achieved through intentional architecture and loss function design in order to accommodate these missing data structures.

Compared to open-source methods, ALPaCA achieves superior segmentations at the lesion level and produces subject-level biomarkers with stronger associations with MS diagnosis. Compared to manual raters, ALPaCA achieves comparable discrimination of subject-wise MS diagnosis while providing voxel-level segmentations and classification of confluent lesions. Though only PRL and CVS identification, in contrast to voxel-wise segmentation, is part of proposed MS diagnostic criteria changes, availability of such segmentations can enable quantitative innovations as well as research insights.

Direct comparisons of ALPaCA to RimNet and CVSnet could not be made as RimNet and CVSnet are not fully automated and could not be run on the CAVS-MS Pilot dataset. However, indirect comparisons could be made using reported metrics from the RimNet and CVSnet manuscripts. The best RimNet model used FLAIR and EPIp images. On the test set, this model reported an AUROC of 0.946 at the lesion level with sensitivity of 70.6% and specificity of 94.9% at an unspecified threshold. This AUROC is higher than that the corresponding ALPaCA AUROC of 0.91 for PRLs. To compare sensitivity and specificity, when the ALPaCA PRL ROC is thresholded such that the sensitivity is approximately 70.6%, the corresponding specificity is 90.6%. Thus, reported RimNet performance may be slightly higher than ALPaCA performance. However, RimNet patch extraction required manually-corrected voxel-wise lesion segmentations in order to center patches based on lesion center of mass and separate confluent lesions. RimNet patch selection also required manual exclusion of lesions near air artifacts and automatic exclusion of lesions with volume over 2746 mm^3^, lesions with volume under 12.3 mm^2^, non-PRL lesions next to PRL lesions, or lesions where part of the lesion segmentation extended outside of a 28 x 28 x 28 voxel patch. This resulted in exclusion of 26% of manually-corrected automatic lesion segmentations from RimNet evaluation—5144 lesions were included, 1671 lesions were excluded due to small size, and 175 lesions were excluded due to other reasons. Such manual data curation steps are likely to result in improved performance. Meanwhile, ALPaCA achieves comparable AUROC, sensitivity, and specificity while allowing for fully-automated PRL segmentation and classification of all lesion candidates without need for manual curation of segmentations or exclusion of challenging lesions. Additionally, RimNet may be less generalizable since it was developed on a two-center dataset while ALPaCA was trained on a ten-center dataset.

CVSnet reported a cross-validated AUROC of 90% with sensitivity of 81% and specificity of 80% at some unspecified threshold. Across its 10 cross-validation folds, CVSnet AUROCs ranged from 84% to 93%. These model performance metrics may be slightly higher than, but overall comparable to, the ALPaCA CVS AUROC of 0.87 with 95% CI from 0.854 to 0.881, Youden’s J sensitivity of 83.6%, and Youden’s J specificity of 74.1%. Like RimNet, CVSnet requires manual interventions in order to curate the candidate lesion dataset. These interventions include manual segmentation of lesion candidates and manual exclusion of lesions not fulfilling NAIMS CVS criteria. Since ALPaCA CVS false positives included a significant number of CVS-excluded lesions, such manual curation would be likely to further improve ALPaCA performance. However, even without manual exclusion of such lesions and using a fully-automated, non-curated approach, ALPaCA is able to achieve comparable performance to CVSnet and is likely more generalizable than CVSnet, which is trained on a three-center dataset. Ultimately, ALPaCA’s architecture and training procedure allow for the refinement of non-curated automated segmentations and individual lesion delineations, allowing for full automation of the overall pipeline.

ALPaCA is trained on highly-augmented, clinically-feasible 3T images from a multi-site dataset that included radiological MS mimics. Thus, ALPaCA predictions may be generalizable across sites for future scans using similar acquisition sequences (Ontaneda et al., 2021). Clinically, the inclusion of MS mimics is particularly relevant since previous studies have shown diagnostic utility of PRLs and CVS in atypical presentations (Absinta et al., 2018). Additionally, lesion segmentations allow for lesion load measurements relevant for treatment decisions (Torkildsen et al., 2016). In research, there is growing interest in quantifying associations between disability and lesion burden, lesion location, and lesion-based network disruption (Baller et al., 2023; Pagani et al., 2020). Automated voxel-wise lesion, PRL, and CVS segmentations may enable neuroscience discoveries, enabling future researchers to study lesion topology, lesion distribution and how distribution relates to both MS diagnosis prediction and underlying pathology.

Methodologically, ALPaCA demonstrates the strengths of data augmentation and pretraining as a form of transfer learning when gold standards are limited (Chlap et al., 2021). Moreover, our architecture incorporates domain-specific knowledge. First, the network shares hidden parameters across three related tasks to increase available information. Additionally, it incorporates known label dependencies by making lesion predictions before PRL and CVS predictions. Such multi-label architectures are beneficial over separately-trained, single-label architectures, but this concept is uncommonly seen in neuroimaging (Ruder, 2017). Finally, ALPaCA uses missingness indicators to account for identifiable missingness in the training data, borrowing concepts from multi-task learning and natural language processing to incorporate all available information, including participants with incomplete labels (Huang et al., 2021; Tardy & Mateus, 2022; Zhang et al., 2021).

While many typical lesions were missed by MIMoSA and therefore ALPaCA, missed lesions occurred largely in non-MS participants. Additionally, the much higher coverage rates of PRLs and CVS in non-MS participants suggest MS-like lesions are still identified in these participants. Missed non-MS-like lesions may limit the lesion segmentation applications of ALPaCA in non-MS settings. However, in the MS setting, missing non-MS-like lesions may ultimately be beneficial for discriminating between MS and non-MS diagnoses. Additionally, APRL and ACVS necessarily missed these same lesions and more since they also rely on MIMoSA to identify lesions.

ALPaCA is a fully automated method for joint segmentation and classification of MS lesions, PRLs, and CVS using clinically-feasible 3T MRI images. ALPaCA integrates modern deep learning concepts to effectively train on data with heterogeneous missing data while accounting for biological dependencies in multi-label predictions. Ultimately, ALPaCA holds great promise in translating PRL and CVS biomarkers to clinical practice as well as in advancing quantitative research studies.

## Supplementary Material

Supplementary Material

## Data Availability

ALPaCA is implemented as an R package (https://github.com/hufengling/ALPaCA). PyTorch and torch for R were used. Development code, including implementation in PyTorch, is available at: https://github.com/hufengling/ALPaCA_analyses. Analyses were conducted using R version 4.1.1 and Python 3.10.5. The dataset will be made publicly available through NAIMS upon completion of CAVS-MS study analysis (Ontaneda et al., 2021).
