# Supplementary material for: Automated segmentation of multiple sclerosis lesions, paramagnetic rims, and central vein sign on MRI provides reliable diagnostic biomarkers
Source: Imaging Neurosci (Camb). 2025 Oct 10;3:IMAG.a.932. doi: 10.1162/IMAG.a.932 (PMC12516162; doi:10.1162/IMAG.a.932)
Supplement: Supplementary Material [file IMAG.a.932_supp.pdf]

5 Supplemental Materials

5.1 Results

5.1.1 Sensitivity analyses

Among the individuals with radiological mimics of MS, six individuals were considered to have RIS, and eight individuals were considered to have CIS. For sensitivity analyses, we present results when these fourteen individuals are either re-classified as having MS or excluded from the analysis. Notably, when individuals with RIS or CIS were re-classified or excluded, only one individual without MS had manually-identified PRLs, leading to large OR and CI estimates.

We ran univariate logistic regression models identical to those in the main analysis, but where individuals with RIS or CIS were re-classified as individuals with MS (Supplemental Figure 1, Top). Corresponding multivariate logistic regression GAMs for manual, ALPaCA, and previous method biomarkers had: degrees of freedom of 8.36, 13.4, and 6.58; AICs of 72.2, 91.3, and 102.3; and had prediction AUROCs of 0.943 (95% CI: [0.900, 0.97]), 0.92 (95% CI: [0.863, 0.974]), and 0.835 (95% CI: [0.752, 0.92]), respectively. As in the primary analysis, DeLong's test showed the manual label model had higher AUROC than the previous method model ($p = 0.015$). Similarly, the ALPaCA model did not have significantly different AUROCs than the manual label or previous methods model ($p = 0.43$, $p = 0.07$).

Finally, we excluded individuals with RIS or CIS from the analysis (Supplemental Figure 1, Bottom). Corresponding multivariate logistic regression GAMs for manual, ALPaCA, and previous method biomarkers had: degrees of freedom of 12.9, 17.4, and 6.8; AICs of 36.1, 64.7, and 83.9; and had prediction AUROCs of 0.997 (95% CI: [0.991, 1.000]), 0.978 (95% CI: [0.951, 1.000]), and 0.863 (95% CI: [0.780, 0.947]), respectively. Note that the AICs in this set of analyses are not directly comparable to previous AICs as fourteen subjects have been excluded. DeLong's test showed the manual label model had higher AUROC than the previous method model ($p = 0.002$) and the ALPaCA model had higher AUROC than the previous method model ($p = 0.01$).

Meanwhile, ALPaCA model did not have significantly different AUROCs than the manual label model ($p = 0.18$). Overall, across the main analysis and sensitivity analyses, consistent results were seen.

5.2 Acknowledgements

We thank the following individuals for their contribution to this manuscript as members of the North American Imaging in MS Cooperative: Enrique Alvarez (University of Colorado, Denver), Moein Amin (Cleveland Clinic Foundation), Christina Azevedo (University of Southern California), Quy Cao (University of Pennsylvania), Eduardo Caverzesi (University of California San Francisco), Bruce Cree (University of California San Francisco), John Derbyshire (National Institutes of Health), Melissa Martin (University of Pennsylvania), Nico Papinutto (University of California San Francisco), Daniel Pelletier (University of Southern California), Vesna Prchkovska (QMENTA Inc, Boston, MA, United States), Marc Ramos (QMENTA Inc, Boston, MA, United States), Praneeta Raza (Cleveland Clinic Foundation), Paulo Rodrigues (QMENTA Inc, Boston, MA, United States), Rohini Samudralwar (University of Texas Health Science Center), Elias Sotirchos (Johns Hopkins University), and Suradech Suthiphosuwan (University of Toronto).

Variable	N	Odds ratio	p
**Manual Lesion Count**	92	0.99 (0.98, 1.00)	0.058
**Manual PRL Score**	92	1.88 (1.35, 3.42)	0.005
**Manual CVS Score**	92	1.11 (1.06, 1.18)	<0.001
**ALPaCA Lesion Count**	92	1.00 (0.99, 1.01)	0.626
**ALPaCA PRL Score**	92	1.35 (1.18, 1.60)	<0.001
**ALPaCA CVS Score**	92	1.16 (1.07, 1.27)	<0.001
**MIMoSA Lesion Count**	92	1.01 (0.99, 1.03)	0.445
**APRL PRL Score**	92	1.27 (1.11, 1.49)	0.001
**ACVS CVS Score**	92	1.04 (1.02, 1.06)	<0.001

Variable	N	Odds ratio	p
**Manual Lesion Count**	78	0.99 (0.98, 1.00)	0.156
**Manual PRL Score**	78	2.24 (1.48, 4.54)	0.004
**Manual CVS Score**	78	1.14 (1.08, 1.22)	<0.001
**ALPaCA Lesion Count**	78	1.00 (1.00, 1.01)	0.275
**ALPaCA PRL Score**	78	1.53 (1.29, 1.93)	<0.001
**ALPaCA CVS Score**	78	1.23 (1.12, 1.39)	<0.001
**MIMoSA Lesion Count**	78	1.01 (1.00, 1.03)	0.180
**APRL PRL Score**	78	1.33 (1.15, 1.58)	<0.001
**ACVS CVS Score**	78	1.05 (1.02, 1.08)	<0.001

Supplemental Figure 1: Forest plot of univariate odds ratios with respect to MS diagnosis in models where age and sex were controlled for. 95% confidence intervals are provided. Odds ratios are shown on a logarithmic axis. Top: Sensitivity analyses where 14 individuals with RIS or CIS are re-classified as having MS. Bottom: Sensitivity analyses where 14 individuals with RIS or CIS are excluded.
